# Rapid implementation of telepharmacy service to improve patient‐centric care and multidisciplinary collaboration across hospitals in a COVID era: A cross‐sectional qualitative study

**DOI:** 10.1002/hsr2.851

**Published:** 2022-10-03

**Authors:** Niloofar Khoshnam‐Rad, Marsa Gholamzadeh, Mehrnaz Asadi Gharabaghi, Shahideh Amini

**Affiliations:** ^1^ Department of Clinical Pharmacy, Faculty of Pharmacy Tehran University of Medical Sciences Tehran Iran; ^2^ PhD Candidate in Medical Informatics, Health Information Management Department Tehran University of Medical Sciences Tehran Iran; ^3^ Department of Pulmonary Medicine, Faculty of Medicine Tehran University of Medical Sciences Tehran Iran

**Keywords:** clinical pharmacy, clinical pharmacy expansion model, COVID‐19 pandemic, critical care, telemedicine, telepharmacy

## Abstract

**Background and Aim:**

The COVID‐19 pandemic forced healthcare systems to apply new technology‐based solutions. The main objective of our study was to describe the conceptual model for rapid implementation of telepharmacy service and the main steps that should be considered.

**Method:**

In response to a limited number of on‐site clinical pharmacy specialists and a lack of technology infrastructure, a cross‐sectional telepharmacy program was established to support major teaching hospitals. A store and forward model of teleconsultation was employed using WhatsApp messenger to cover various aspects of multidisciplinary collaboration in COVID‐19 management. All identifiable personal information was removed from all exchanged messages of collaborative consultations. The thematic analysis of consultations was performed to extract the main themes and subthemes that should be considered for designing future telepharmacy systems.

**Results:**

Through telepharmacy service, 600 intensive care unit teleconsultations for COVID‐19 cases were conducted in the residence center and nonresidence centers. In total, 1200 messages were exchanged between specialists in 3 months. The average time taken to respond to a message was 1.30 h. Thematic analysis revealed four main concepts and 15 subconcepts that should be considered in telepharmacy consultations for COVID‐19 management. Based on the extracted themes, a conceptual model for developing a telepharmacy program was devised.

**Conclusion:**

The results showed that by utilizing telehealth, clinical pharmacists could cover critically ill patients who need pharmacotherapy counseling through interdisciplinary collaboration. Moreover, the main features of our service that are represented through this survey can be employed by other researchers for developing telepharmacy services.

## 1 BACKGROUND

COVID‐19 is a complex multisystem disorder, severe cases of which may require intensive care unit (ICU) admission. The rapid increase in COVID‐19 cases led to significant demand for hospital admissions and intensive care.^1^, ^2^ During the pandemic, healthcare professionals utilized different solutions to ensure the optimal management of infected patients with the virus and those at risk of the infection.^3^ To improve the efficiency of patient care, interprofessional and multidisciplinary collaboration across hospitals and health systems is augmented during a pandemic.^4^ Due to the high ambiguity of inpatient treatment, clinical pharmacists have a crucial role in healthcare team practices^5^ as active members of the interdisciplinary critical care team.^6^

During the COVID‐19 pandemic, several drugs were used in the context of clinical trials or for off‐label use; and many physicians were unfamiliar with them. Thus, patients required comprehensive medical management with clinical pharmacist consultation to guide physicians and ensure the safe and effective usage of drugs. Pharmacists' participation in clinical decision‐making facilitates COVID‐19 management according to the last clinical protocols.^7^

The lack of critical care pharmacists and intensivists was the greatest obstacle to providing appropriate pharmacotherapeutic care in critical care units. In addition, interactions between specialists in clinical rounds have been restricted due to close contacts limitation during the pandemic.^2^ To overcome the mentioned obstacles,^8^ telemedicine‐based, and innovative approaches were taken by researchers to minimize these barriers to interprofessional collaboration.^9^ It was an opportunity to apply technology at a speed that would not be possible at any other time to reduce the risk of exposure.^10^

Telepharmacy is one of the telemedicine aspects that can provide near real‐time consultation around the country and meet the need to access high‐quality and up‐to‐date evidence.^11^, ^12^ COVID‐19 has also resulted in overloaded, ever‐changing information. Nearly 8000 articles were published in the first 4 months of the outbreak. This abundance of information contributes to variability in practice, confusion, and decision fatigue which can affect the ability of healthcare providers to provide optimal patient care.^13^, ^14^ The information overload and the complexity of COVID‐19 cases made it even more essential to work and decide together to serve patients. As the subject of medicine is so broad, the collaboration between multidisciplinary team (MDT) members provides a multidimensional thinking pattern; this can enhance ease of decision‐making and improve patient safety.^15^ The present pandemic provided an opportunity to feel the value of each member of the MDT.

Using telepharmacy in the COVID‐19 pandemic can improve consultation with other healthcare providers and decrease adverse drug effects and interactions.^16^ Thus, clinical pharmacists can expand their services to more patients in different centers. Telepharmacy can eliminate the need for on‐site pharmacists in a clinical setting for consultation.^12^ However, it needs a platform to connect healthcare providers with pharmacists to share their information and communicate with others. A wide variety of free chat‐based software is currently available (like WhatsApp, Viber, Google Hangouts, Zoom, Telegram, and Skype) with good camera quality and a good internet connection.^17^ They allow instant sharing of the necessary information between healthcare professionals. Messaging applications like WhatsApp Messenger are using end‐to‐end encryption that enhances the security of shared information.^18^ Due to WhatsApp messenger's popularity among healthcare providers and freely available for most smartphones with different operating systems, it was selected as an appropriate tool to implement telepharmacy service as soon as possible.

The current pandemic has forced individuals and healthcare systems to review feasible and desirable models to manage this crisis and changed previous practices. Our main goal is to quickly implement a free online consultation service to access pharmaceutical care in the difficult circumstances of the pandemic. The main aim of our study was to describe an easy and fast solution to improve interprofessional collaboration between different specialists in hospitals for the management of critically ill patients through telemedicine. Other objectives of our study were to describe a conceptual model for developing telepharmacy services to expand clinical pharmacy consultation in a time of crisis through qualitative analysis.

## 2 METHODS

Following the global outbreak of COVID‐19 and two deaths in Qom city due to COVID infection, the Iranian Ministry of Health officially announced the COVID‐19 outbreak in the country. Over a short period (February 19 to March 11), Iran became one of the most affected countries in the world. The pharmacotherapeutic care of these patients who required intensive care was complex.^19^ As COVID‐19 spreads across Tehran, on February 18, 2020, in response to a limited number of on‐site clinical pharmacy specialists, a cross‐sectional telepharmacy program was established in Shariati and Imam Khomeini hospitals which are the two main teaching hospitals of Tehran University of Medical Sciences. We employed a store‐and‐forward model of teleconsultation using WhatsApp messenger to facilitate teamwork collaboration. Store and forward referred to a type of asynchronous telemedicine service in which clinical data were collected, captured, and transformed electronically. Store‐and‐forward services provide access to data after it has been collected outside of real‐time patient interaction.^20^, ^21^ CAT Scans, magnetic resonance imaging, X‐rays, photos, videos, and text‐based patient data are gathered and sent to clinical pharmacists using WhatsApp after de‐identifying personal information.^22^, ^23^ The method and results were reported according to the Consolidated criteria for reporting qualitative research checklist for qualitative studies.

### 2.1 Inclusion and exclusion criteria

Briefly, participants were physicians treating COVID‐19 cases that were admitted to the ICU. All of them were employed by the teaching hospitals of Tehran University of Medical Sciences. Participants were included in this study if they had a valid WhatsApp account and if they agreed to use WhatsApp for consultations. According to our protocol, if their patients need a consultation with a clinical pharmacist, they use WhatsApp to consult clinical pharmacists. All COVID‐19 patients who needed teleconsultations were eligible for our study and included in the study when the clinical pharmacist received the first message of consultation request. We analyzed the data regarding these consultations. Physician's participation was also voluntary and anonymity was guaranteed. In this study, patients whose consultations were related to other diseases and patients afflicted with problems involving multiple services except intensive care were excluded from the study. Eligible patients who were treated by an intensivist in consultation with a clinical pharmacist at the bedside via WhatsApp platform were evaluated in the study.

### 2.2 Designing telepharmacy service

Due to busy working conditions, lack of clinical pharmacy specialists, and the need for transportation, real‐time consultations could not be performed at the patient's bedside during the COVID‐19 pandemic. Following these circumstances, consultations via WhatsApp emerged naturally due to the overcrowding of ICU patients.

#### 2.2.1 WhatsApp platform

WhatsApp© (WhatsApp Inc.) is an instant messaging application for smartphones using the Internet connection to send different types of data, including text messages, images, video, user location, and audio messages. We utilized the WhatsApp platform for sharing information due to its popularity, free‐of‐charge accessibility, and ease of use. This selection was made for the rapid implementation of the program during the COVID pandemic and lack of clinical resources condition. Nevertheless, any social networking platform can be used according to the experts’ demands as far as confidentiality could be maintained.

The WhatsApp version 2.10 or higher was employed in this survey for real‐time consultation. The WhatsApp numbers used for teleconsultation were synced and linked to the web application form of WhatsApp to increase accessibility and make consultation more coinvent.

The interventions performed by the clinical pharmacist via telepharmacy cover various aspects of the COVID‐19 management process. The teleconsultations took place between pharmacists and corresponding physicians on the WhatsApp platform. None of the referred cases were excluded. After performing point‐of‐care examinations and completing laboratory test results and imaging reports, consultation with a pharmacist was accomplished. In addition to the lab test results, the outcomes of physical examinations, clinical assessments, and disease progression notes were sent to the clinical pharmacist for consultation by intensive care physicians. Then, anonymous clinical reports were forwarded to the clinical pharmacist to suggest the best treatment plan. Due to the complexity of COVID‐19 disease, the patient admitted to the ICU requires long‐term care and various counseling. Hence, multiple consultations were made possible for each patient within a day. Implied consent was obtained for any medical intervention and sharing of information at the beginning of treatment for patients or their families in the critical care unit. According to Mars et al.,^24^ the processing of personal data for full anonymization is “compatible with the purpose for which the personal data are initially collected” and therefore does not require an additional legal basis, such as consent, specifically for the act of anonymizing.

After evaluating the consultation messages, the clinical pharmacist's suggestions were sent as voice or text messages. All consultations were done without patients' identification data. Participants were asked to disable the backup option in WhatsApp settings. Real‐time consultations were conducted based on Barayev's^25^ study to overcome WhatsApp security problems. After reviewing the messages related to the consultations, one of the researchers provided the relevant content to the analyst and the participants were requested to delete the messages from their phone's memory for security reasons.

The moment of sending the consultation request was recorded as the beginning of the patient's consultation period. Accordingly, the response time to the WhatsApp request was assumed as the consultation response time.

### 2.3 Analysis of results

After 3 months of conducting the telepharmacy program in our clinical pharmacy service, the content of the consultations was converted to Word documents for qualitative analysis. A thematic analysis^26^ was applied to determine the main domains, categories, and information flow of the telepharmacy service. Messages were examined in the context of medication advice that was posted. The process of identifying themes was conducted iteratively according to Braun and Clarke's^26^ approach.^27^ This made it possible to identify the most common issues that were discussed through consultation. Each message was read several times to generate a list of potential themes. Themes and sub‐themes were classified to form the conceptual framework of telepharmacy service.

For ethical issues, all of the extracted messages were deidentified. In this study, only descriptive‐analytic was done with no analytical statistics. Consequently, the authors represent a conceptual model for developing a telepharmacy program to fight with limitations caused by the COVID‐19 pandemic.

## 3 RESULTS

At the time of intervention, our team participated simultaneously in the treatment team of two different centers (10 and 13 ICU beds). Through telepharmacy service, 200 probable and confirmed ICU teleconsultations for COVID‐19 cases were conducted in the residence center, in addition to 400 patients were consulted in non‐residence centers. All included patients were diagnosed with COVID‐19 and hospitalized in ICUs at the first peak of the pandemic. Before the pandemic, a clinical pharmacist attended a team‐based round which it lasts 4 h a day, three times a week. During the COVID‐19 pandemic, telepharmacy consultations have been added to routine team‐based care. In total, 1200 messages were exchanged between individuals during this study. The average time taken to respond to the request on the WhatsApp platform was 1.30 h (range: 0.50–4 h).

### 3.1 Service description

**Figure 1:**
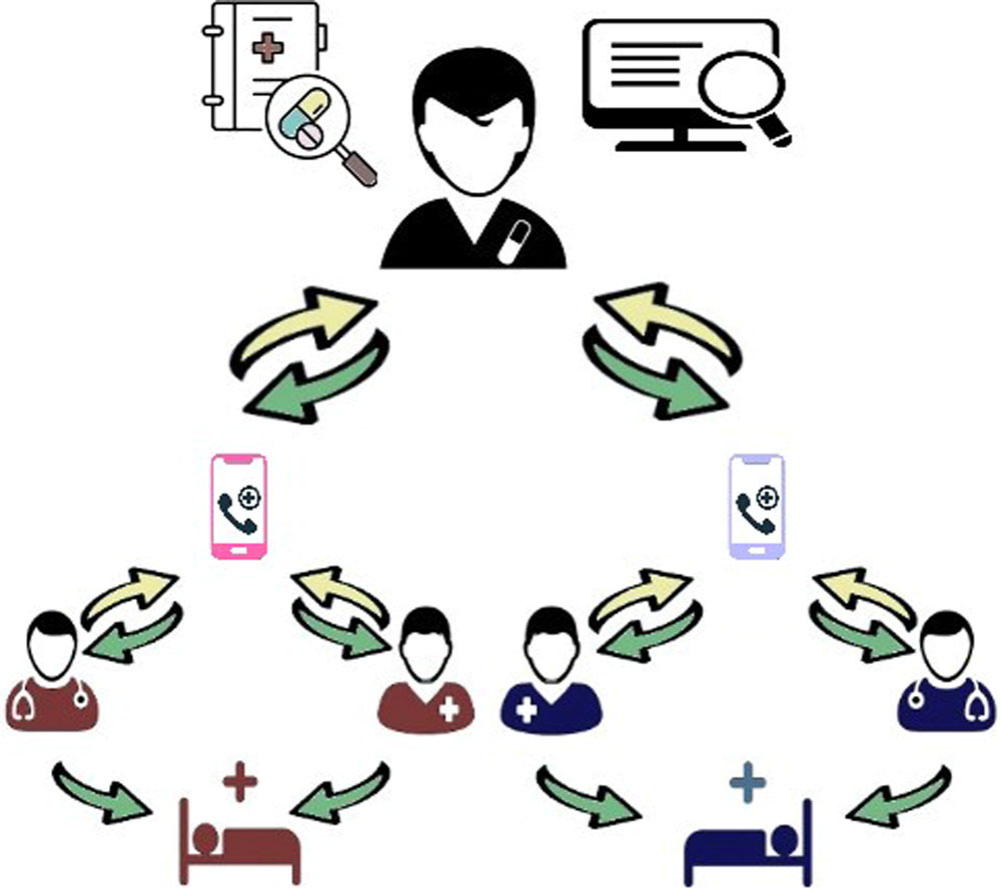
The telepharmacy consultation model (In this model, physicians and nurses from two different hospitals [red and blue teams]) can share clinical information and patient records (yellow arrows) with a clinical pharmacist through telemedicine tools. After careful evaluation of the case and reviewing the latest information, the appropriate medication management considering the patient's condition (green arrows) will be shared by the clinical pharmacist, and the patient benefits from it

The general model of implemented telepharmacy service in our center and the interaction between MDT members are described in Figure 1. Through our survey, we found that a clinical pharmacist can cover at least two wards through telepharmacy. In the proposed model, the wards are covered by the clinical pharmacist in two different hospitals which are in a similar situation (e.g., two respiratory wards in two different hospitals). In this model, physicians and nurses from two different hospitals (red and blue teams) can share clinical information and patient records (yellow arrows) with a clinical pharmacist through telemedicine tools.

After careful evaluation of the cases and reviewing the latest information, the appropriate medication management considering the patient's condition (green arrows) will be shared by the clinical pharmacist. These messages included not only text‐based messages but also image‐based and audio messages. Image‐based messages included radiology findings, lab data, and patient medication lists. In the following, the models and frameworks were obtained through consultations analysis were represented.

### 3.2 Different main aspects of critical care clinical pharmacist interventions

**Figure 2:**
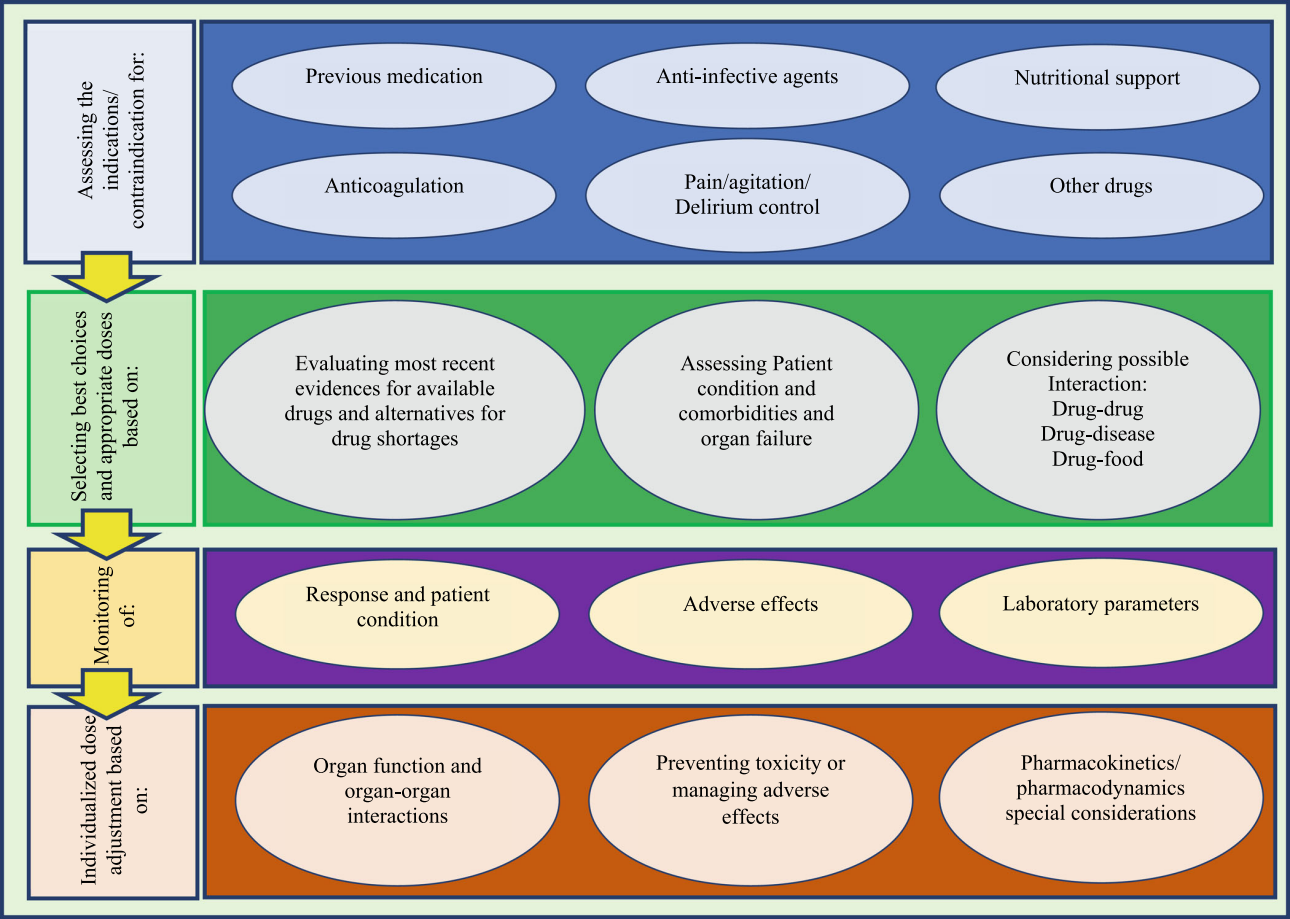
Themes and subthemes of clinical pharmacist interventions to ensure the safe and effective use of drugs. Themes are shown on left in rectangular shapes, and subthemes are shown on right in oval shapes

Thematic analysis of conducted message‐based consultations revealed that telepharmacy domains can be categorized into four main themes and 15 sub‐themes. Themes are shown in Figure 2 in the form of rectangular shapes. The main themes represent the key steps that should be considered for the management of COVID patients to conduct a remote clinical pharmacist consultation.

These four key steps (themes) are, (1) Assessing indication or contraindication in six main domains, (2) Selecting best choices and appropriate doses based on three main domains, (3) Monitoring patients based on three main domains, and (4) Individualized dose adjustment based on three main criteria. Sub‐themes that should be considered in each step are represented in oval shapes in each phase. The themes and subthemes in Figure 2 can be used as a conceptual model to determine the characteristics of a systematic remote drug consultation system.

These extracted themes showed that telepharmacy consultations covered a wide variety of problems, including antimicrobial management, individualized therapeutic adjustment, anticoagulation, nutrition support, pain, agitation, and delirium management. Other sub‐themes covered the safety and effectiveness of drugs and medication plans. The content of the advice exchanged in the form of messages between healthcare providers is given in Table 1 as examples of each category for better understanding.

**Table 1:** Examples of different scopes of interventions of critical care pharmacists in the COVID‐19 pandemic in our center

Scope of the intervention	Examples
COVID‐19 management	Providing and updating information about the appropriate use of repurposed drugs for COVID‐19
	Providing and updating information about disinfectants
	Evaluating indications or contraindications to repurposed drugs for each patient
	Providing information about the appropriate use of anticoagulants in COVID‐19
	Acting as principal investigator or coinvestigator of research teams for conducting clinical trials
	Deciding about symptomatic treatment (cough, diarrhea, fever, etc.)
Nutritional support	Estimating required daily calories for patients and deciding about pharmaco‐nutrition and source of nutrition support
	Providing consultation about the enteral or parenteral route of nutrition
	Consulting about the possible interaction between nutritional support and prescribed drugs
	Consulting about drug administration via nasogastric tube
	Providing information about vitamin and mineral supplementation in the management of COVID‐19
Pain, agitation, and delirium management	‐ Deciding on the selection of appropriate analgesics, anxiolytics, or antipsychotics
	‐ Management of side effects of analgesics, anxiolytics, or antipsychotics
	‐ Management of drug‐drug interactions
	‐ Differentiating drug side effects (e.g., respiratory depression, tachycardia) with symptoms of COVID‐19 disease or anxiety
	‐ Appropriate use of opioids for the management of cough besides the pain control
Antimicrobial management	Providing information about the appropriate use of repurposed antibiotics for COVID‐19
	Preventing antibiotic misuse and overuse
	Conduct and translate research on all areas of antimicrobial resistance and antimicrobial stewardship
	Management of side effects and interactions
	Providing solutions for drug shortage
	Therapeutic drug monitoring and appropriate dose adjustment
	Appropriate drug for super‐infections and co‐infections
	Evaluating efficacy and toxicities
Individualized therapeutic adjustment	‐ Evaluating kidney and liver function and adjusting the dose of drugs accordingly
	‐ Providing plans for preventing toxicity in patients with multiorgan failure
	‐ Dose adjustment based on augmented renal clearance in septic patients
	‐ Dose adjustment or drug discontinuation in case of side effects
	‐ Drug dosing in special populations (elderly, obesity, pregnancy, pediatrics, …)
Anticoagulation	Assessing thromboprophylaxis for patients
	Monitoring of anticoagulants efficacy
	Managing side effects and interactions related to anticoagulation
	Assessing the indication for stress ulcer prophylaxis
	Assessing the indications and contraindications for the use of different anticoagulants, including direct oral anticoagulants

### 3.3 Sharing information and decision‐making process in the telepharmacy program

**Figure 3:**
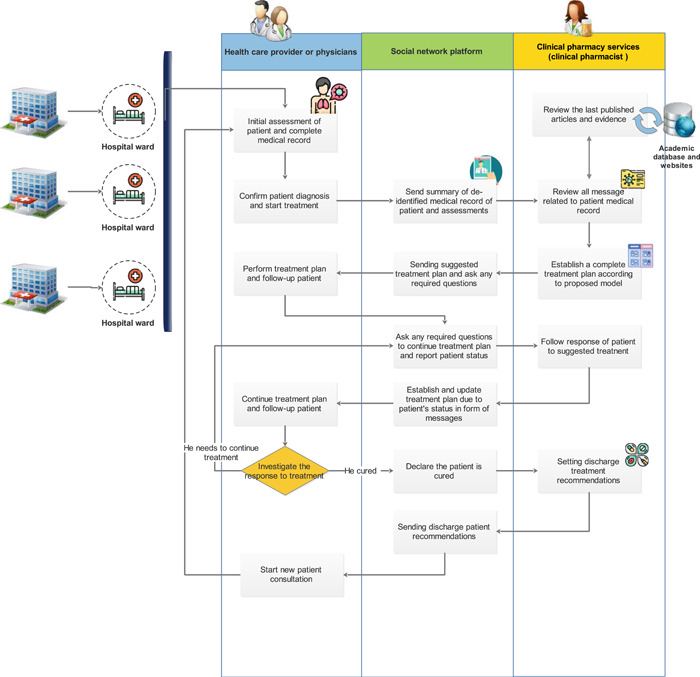
The clinical decision‐making and sharing of information flow

As we showed in the proposed model of applying telemedicine, pharmacists can play a more active role in increasing access to the latest evidence and protocols for the medical care team. Based on conducted consultation, we proposed a model in Figure 3 to represent a set of tasks that occurred during telepharmacy service establishment. Through this model, we can find out how clinical pharmacists can communicate with several centers through teleconsultation. The information in the proposed model refers to any types of patients’ clinical data that are collected during pharmacotherapeutic care (Table 2). Additionally, scientific information in the form of protocols and practice guidelines can be shared between several centers.

**Table 2:** Information that can be shared between different hospitals

Information about new drugs indications for COVID‐19
Protocols for appropriate and rational use of drugs
Protocols for the administration of drugs
Protocols for managing common drug‐related problems (ADR, drug‐drug interactions, …)
Patient education materials

## 4 DISCUSSION

The COVID‐19 pandemic has affected the world in many ways and forced health systems to rapidly adopt telemedicine‐based solutions to improve patient‐centered care. Through this study, we established telepharmacy care and suggested a conceptual model addressing the overloaded pharmacy services during the COVID‐19 pandemic. Our research showed that the telepharmacy service was convenient, and it can provide high‐quality, rapid, and time‐saving consultations with other healthcare providers.

The proposed model could offer a feasible and cost‐effective way to expand the clinical pharmacists’ services, but it might encounter a few obstacles. As the main objective of our study was to provide a model and determine the main components of telepharmacy service in the context of teleconsultation for implementing a third‐party system in further study, we discuss some of the possible challenges and benefits of our proposed model.

Our survey showed that telepharmacy service has three main components: (1) a pharmacist who is the key member in a telemedicine service who provides consulting services to applicants, (2) a social media application or an electronic chat‐based software platform that provides sharing information, the possibility of conversation and consultation between experts, and (3) the specialists who seek medical consult.

Though pharmacists are recognized as essential members of the critical care team, the pharmacist‐to‐patient ratio is even low in developed countries.^6^, ^28^ Thus, a telepharmacy service is implemented to provide this opportunity for clinical pharmacy service to support more patients with extra work in special situations like a pandemic.

Telemedicine services have become an integral part of medicine.^9^ Evidence has shown that social media has a pivotal role in teleconsultations.^17^ In this study, WhatsApp messenger was selected for rapid implementation. In this study, WhatsApp messenger, a free‐of‐charge messaging platform that can provide sharing instant messages with pictures and videos, was selected based on our objectives.^25^, ^29^ However, the suggested model and determining essential components represented through this research could help specialists to develop their telepharmacy system with other software.

The suggested telepharmacy model provides telework for consultation which means the ability to work from home or anywhere to save more time. However, the high volume of cases and their complexity leaves very little extra time for any other work. Although our results show that pharmacist counseling could expand through telepharmacy to more patients, more time should be devoted even out of business hours. In our program, critical care clinical pharmacists must balance a high patient care volume with significant other responsibilities, including leadership roles, precepting learners, and quality improvement. Some of their duties should be delegated to others, including pharmacists, pharmacy technicians, or students. But this delegation is not always possible and efficient, even in such a telemedicine‐based program.

The security of personal health data transmitted electronically is always a concern of telehealth programs. Since there is no ready infrastructure for teleconsultation and the pandemic forced us to act rapidly, we used WhatsApp messenger, which uses end‐to‐end encryption that enhances the security of shared information.

For proper patient care, those directly involved in patient care must be able to share patient information to coordinate care, and this is not against the principles of confidentiality.^24^, ^30^ In general, medical information and patients’ data should only be shared with those involved in patients’ care. Hence, the healthcare institution does not need to ask the patient repeatedly for disclosure of PHI (personal health information) in the routine care process.^31^ However, WhatsApp in its current form was noncompliance with HIPPA (Health Insurance Portability and Accountability Act), only the deidentified results of clinical tests and medical history of patients were sent to team members through our service to adhere to the principles of confidentiality. Health information is “deidentified” by the HIPAA privacy rules.^32^, ^33^ On the other hand, circumstances may occur in which the patient cannot be informed of the disclosure of personal information, for example in a pandemic situation where the patient's life is at risk. In such cases, we must pass on the relevant information promptly to those providing the patient's care to save the patient's life.^34^

The clinical pharmacy specialists were not extra paid for their participation in our implemented program. Hence, it was implemented only to improve patients’ health. Due to the low number of clinical pharmacists, not all university‐affiliated hospitals have sufficient clinical pharmacists to cover all the consultations. However, clinical pharmacists hold themselves accountable to all patients admitted to university hospitals. Therefore, although there was no direct payment for consultations in other hospitals, all clinical pharmacists were employed by the university and were responsible for helping in the special situation of the pandemic.

Although our teleconsultation services are nonprofit and it was free of charge, determining the charge of teleconsultations is one of the main challenges for the health system. We utilized a free platform to address this challenge. To implement digital health successfully, hospital managers, insurance companies, and policymakers should understand the financial efficiencies of telehealth services and decide how the various payment methodologies can impact the growth of healthcare telehealth technologies and innovations.^23^

## 5 CONCLUSION

During the complexity and chaos of COVID‐19, considering strategies for improving team‐based approaches to providing high‐quality patient care is regarded as a critical need. Here, concerning insufficient infrastructure, we report the simple method to explain how telemedicine clinical pharmacy consultation and collaboration were implanted to provide personalized management care during Infodemia (abundance of accurate and inaccurate information) of COVID‐19. The represented model in this paper can be used as an effective roadmap for the rapid implementation of remote consulting in the absence of proper infrastructure in the face of a crisis. The results showed that by utilizing telehealth, clinical pharmacists could cover critically ill patients who need pharmacotherapy counseling through interdisciplinary collaboration. Moreover, the main features of our strategy represented through this survey can be employed by other researchers for developing telepharmacy consultation systems.

## AUTHOR CONTRIBUTIONS

**Niloofar Khoshnam‐Rad**: conceptualization; data curation; formal analysis; investigation; methodology; supervision; validation; visualization; writing—original draft; writing—review & editing. **Marsa Gholamzadeh**: conceptualization; formal analysis; investigation; methodology; supervision; validation; visualization; writing—original draft; writing—review & editing. **Mehrnaz Asadi Gharabaghi**: conceptualization; data curation; investigation; methodology; supervision; validation; writing—original draft. **Shahideh Amini**: conceptualization; data curation; formal analysis; investigation; methodology; project administration; supervision; validation; visualization; writing—original draft; writing—review & editing.

## CONFLICTS OF INTERESTS

The authors declare no conflicts of interest.

## ETHICS STATEMENT

The authors affirm that this manuscript is an honest, accurate, and transparent account of the study being reported; that no important aspects of the study have been omitted; and that any discrepancies from the study as planned (and, if relevant, registered) have been explained.

## Data Availability

The data that support the findings of this study are available on request from the corresponding author.
